# Functional effects of spinocerebellar ataxia type 13 mutations are conserved in zebrafish Kv3.3 channels

**DOI:** 10.1186/1471-2202-11-99

**Published:** 2010-08-16

**Authors:** Allan F Mock, Jessica L Richardson, Jui-Yi Hsieh, Gina Rinetti, Diane M Papazian

**Affiliations:** 1 Department of Physiology David Geffen School of Medicine, University of California at Los Angeles, Los Angeles, California 90095-1751 USA; 2 Molecular Biology Institute University of California at Los Angeles, Los Angeles, California 90095-1570 USA; 3 Brain Research Institute University of California at Los Angeles, Los Angeles, California 90095-1761 USA

## Abstract

**Background:**

The zebrafish has been suggested as a model system for studying human diseases that affect nervous system function and motor output. However, few of the ion channels that control neuronal activity in zebrafish have been characterized. Here, we have identified zebrafish orthologs of voltage-dependent Kv3 (KCNC) K^+ ^channels. Kv3 channels have specialized gating properties that facilitate high-frequency, repetitive firing in fast-spiking neurons. Mutations in human Kv3.3 cause spinocerebellar ataxia type 13 (SCA13), an autosomal dominant genetic disease that exists in distinct neurodevelopmental and neurodegenerative forms. To assess the potential usefulness of the zebrafish as a model system for SCA13, we have characterized the functional properties of zebrafish Kv3.3 channels with and without mutations analogous to those that cause SCA13.

**Results:**

The zebrafish genome (release Zv8) contains six Kv3 family members including two Kv3.1 genes (*kcnc1a *and *kcnc1b*), one Kv3.2 gene (*kcnc2*), two Kv3.3 genes (*kcnc3a *and *kcnc3b*), and one Kv3.4 gene (*kcnc4*). Both Kv3.3 genes are expressed during early development. Zebrafish Kv3.3 channels exhibit strong functional and structural homology with mammalian Kv3.3 channels. Zebrafish Kv3.3 activates over a depolarized voltage range and deactivates rapidly. An amino-terminal extension mediates fast, N-type inactivation. The *kcnc3a *gene is alternatively spliced, generating variant carboxyl-terminal sequences. The R335H mutation in the S4 transmembrane segment, analogous to the SCA13 mutation R420H, eliminates functional expression. When co-expressed with wild type, R335H subunits suppress Kv3.3 activity by a dominant negative mechanism. The F363L mutation in the S5 transmembrane segment, analogous to the SCA13 mutation F448L, alters channel gating. F363L shifts the voltage range for activation in the hyperpolarized direction and dramatically slows deactivation.

**Conclusions:**

The functional properties of zebrafish Kv3.3 channels are consistent with a role in facilitating fast, repetitive firing of action potentials in neurons. The functional effects of SCA13 mutations are well conserved between human and zebrafish Kv3.3 channels. The high degree of homology between human and zebrafish Kv3.3 channels suggests that the zebrafish will be a useful model system for studying pathogenic mechanisms in SCA13.

## Background

Voltage-dependent Kv3 K^+ ^channels have specialized gating properties, including a depolarized activation range, fast activation, and very fast deactivation, that facilitate rapid, repetitive firing in neurons [1,2]. In mammals, there are four Kv3 genes, *KCNC1*-*KCNC4*, which encode Kv3.1-Kv3.4 [3]. Each gene is alternatively spliced, generating channel proteins with different carboxyl-terminal sequences [1]. Kv3.3 and Kv3.4 contain amino-terminal extensions that mediate N-type ball-and-chain inactivation [1].

Recently, *KCNC3*, which encodes Kv3.3, was identified as the gene mutated in spinocerebellar ataxia type 13 (SCA13) [4,5]. The spinocerebellar ataxias are a group of 28 human autosomal dominant genetic diseases characterized by motor deficits, eye movement abnormalities, and degeneration of cerebellar neurons [6,7]. SCA13 is the first neurodegenerative disease known to be caused by mutations in a K^+ ^channel gene [4].

The two originally-identified SCA13 mutations lead to distinct clinical manifestations that are likely caused by their differential effects on Kv3.3 function [4]. The R420H mutation is associated with adult onset, progressive ataxia accompanied by progressive cerebellar degeneration. R420H is located in the S4 transmembrane segment, the main functional element of the voltage sensor. This mutation suppresses the amplitude of Kv3 currents by a dominant negative mechanism [4]. In contrast, the F448L mutation is associated with persistent motor deficits that emerge in infancy. In affected children, the cerebellum is severely shrunken and malformed [8]. F448L is located near the cytoplasmic end of the S5 transmembrane segment, a region of the protein that couples voltage sensor conformational changes to opening and closing of the pore [9]. This mutation affects the unique gating properties of Kv3 channels, shifting the voltage dependence of pore opening in the hyperpolarized direction and dramatically slowing channel closure [4]. Interestingly, F448L changes a phenylalanine residue found only in Kv3 channels to leucine, the residue found at the analogous position in all other Kv channel subfamilies [3]. As a result, the mutation confers Shaker-like gating properties on Kv3.3 [4]. The distinct clinical manifestations of the R420H and F448L mutations are not likely to result from differences in genetic background because there is a strong genotype/phenotype correlation for age of disease onset in unrelated SCA13 families [5].

Kv3.3 is prominently expressed in cerebellar neurons [10,11]. Given the importance of Kv3 channels in controlling neuronal firing patterns, the locomotor deficits and loss of cerebellar neurons seen in SCA13 may result from changes in the excitability of Kv3.3-expressing cells. Development of an animal model is essential to investigate the mechanistic basis of SCA13 and to explore the connections between electrical excitability, control of locomotor behavior, and neuronal cell death.

In recent years, the zebrafish, *Danio rerio*, has been used extensively to investigate neuronal development. In addition, work from a growing number of laboratories demonstrates that zebrafish has great potential for analyzing nervous system function [12-16]. The zebrafish has been suggested as a model system for studying diseases that affect neuronal function and locomotion [17-19]. As the first step in assessing the suitability of zebrafish as a model system for SCA13, we have identified Kv3 family members in zebrafish and characterized the functional properties of wild type and mutant Kv3.3 channels.

We report that the zebrafish genome (Zv8) encodes six Kv3 family members including two Kv3.3 genes, *kcnc3a *and *kcnc3b*. Zebrafish and mammalian Kv3.3 channels exhibit strong functional homology and are similarly affected by SCA13 mutations. These results suggest that the zebrafish is a promising model system for investigating the pathogenic mechanisms underlying SCA13.

## Results and discussion

### The zebrafish genome contains six Kv3 family orthologs

To identify members of the *KCNC *gene family in zebrafish, the Zv8 genome release was queried with multiple conserved segments of mammalian Kv3 protein sequences using the program Tblastn. Sequences with the highest scoring similarity to mammalian Kv3 channels consistently mapped to six genomic locations (Table 1). In contrast, lower scoring hits showed greater similarity to members of other Kv subfamilies (data not shown).

**Table 1:** Genomic locations of Kv3 family orthologs in zebrafish genome (Zv8)

*Chromosome*	***Identity***^*1*^	*Strand*	***N-term. Exon***^***2***^	***S1-S6 Exon***^***2***^	***Prox. C-term. Exon***^***2***^	***Distal C Exon***^***2***^	***Alt. C Exon***^***2***^
7	*kcnc1a*	+	33,357,526	33,399,062	33,413,557	33,416,890	33,417,909
25	*kcnc1b*	-	4,398,437	4,372,686	4,369,562	4,367,934	
4**^3^**	*kcnc2*	-	2,653,160	2,619,906	2,617,395	2,614,222	
3	*kcnc3a*	+	29,217,143	29,277,151	29,286,001	29,288,301	29,305,269
24	*kcnc3b*	-	*35,119,437***^4^**	35,441,453	35,436,978	35,394,285(+)**^5^**	
8	*kcnc4*	-	25,733,068	25,708,221	25,706,162	25,696,460	

^1 ^Identities were assigned on the basis of the phylogenetic and syntenic analyses shown in Figs. 1-3.

^2 ^The identified exons encode the N-terminus, the S1-S6 membrane domain, the proximal portion of the C-terminus, and one or more putative sequences for the distal C-terminus, which may be alternatively spliced. The numbers correspond to the chromosomal positions of each exon.

^3 ^According to Zv8, there is an additional exon encoding a Kv3 N-terminus on chromosome 4 (minus strand) at position 2,762,001. Because previous genome releases contained stray Kv3 exons that disappeared in subsequent releases, this exon is likely to reflect an assembly error (data not shown).

^4 ^In Zv8, the N-terminal exon of kcnc3b (italics) is out of order compared to the other exons.

^5 ^In Zv8, the distal C-terminal exon of kcnc3b has been mapped to the plus strand, in contrast to the other exons.

To assemble putative sequences for Kv3 proteins in zebrafish, predicted coding exons were identified directly by sequence similarity to the mammalian Kv3 proteins. Exons encoding the amino terminus, transmembrane core domain, and proximal carboxyl terminus were found at each of the six genomic locations (Table 1). Many of these exons were also recognized by Ensembl transcript identification algorithms. In addition, each location contained one or more exons encoding the distal carboxyl terminus, a region that is alternatively spliced in mammalian Kv3 genes (Table 1).

### Phylogenetic identification of Kv3 orthologs in zebrafish

**Figure 1:**
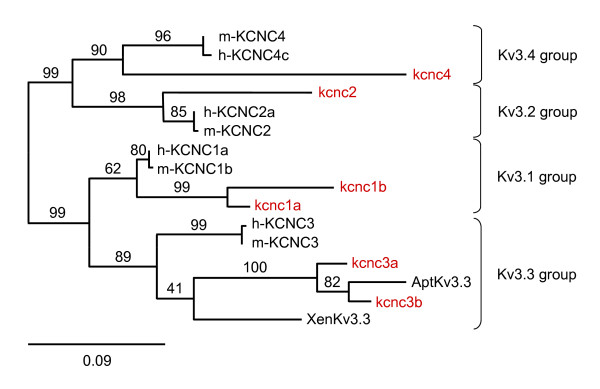
**Identity of Kv3 family members in zebrafish**. Two Kv3.3 (*kcnc3a*, *kcnc3b*), two Kv3.1 (*kcnc1a*, *kcnc1b*), and one each Kv3.2 (*kcnc2*) and Kv3.4 (*kcnc4*) genes were identified using the program PhyML [22,23]. Zebrafish sequences (labeled in red) were aligned with Kv3 sequences from the following species: h, human; m, mouse; Xen, *Xenopus laevis*; Apt, *Apteronotus leptorhynchus *(another teleost fish). The alignment and accession numbers for each non-zebrafish sequence are provided in additional file 1, Fig. S1. Numbers on the tree indicate the percentage of trees that contained the labeled node. The scale bar indicates 0.09 substitutions per amino acid residue.

For phylogenetic analysis of the six putative Kv3 genes in zebrafish, exon sequences encoding the amino terminus, transmembrane core domain, and proximal carboxyl terminus were assembled and translated. Sequences encoding probable alternatively-spliced, distal carboxyl termini were not included. The deduced protein sequences were aligned with ten Kv3 sequences from mammalian, amphibian, and teleost species using the program MUSCLE v3.7 and the alignment was manually adjusted (Additional file 1, Fig. S1) [20,21]. The identities of zebrafish Kv3 genes were assigned by bootstrap analysis replicated 100 times using the program PhyML (Fig. 1) [22,23]. The zebrafish genome contains two *KCNC1 *(Kv3.1) orthologs, designated *kcnc1a *and *kcnc1b*; two *KCNC3 *(Kv3.3) orthologs, designated *kcnc3a *and *kcnc3b*; and one ortholog each of *KCNC2 *(Kv3.2) and *KCNC4 *(Kv3.4) designated *kcnc2 *and *kcnc4*, respectively (Fig. 1, Table 1). Zebrafish Kv3.3 and Kv3.4 sequences contained N-terminal extensions as found in their mammalian counterparts (Additional file 1, Fig. S1) [1]. Over the aligned region, the amino acid identity between human Kv3 sequences and their zebrafish orthologs ranged from 64 to 82% (Additional file 1, Fig. S1).

**Figure 2:**
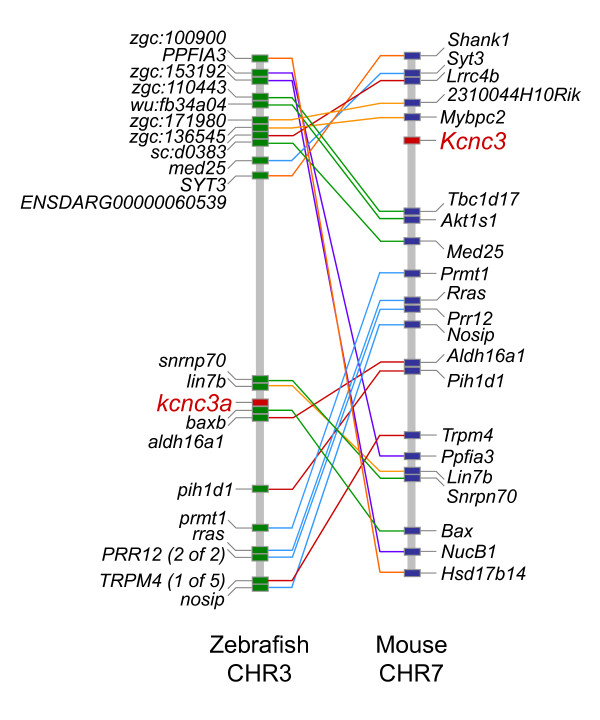
**Synteny between *kcnc3a *on zebrafish chromosome 3 and *Kcnc3 *on mouse chromosome 7**. Using Synteny Database, synteny in the vicinity of Kv3.3 genes was analyzed using zebrafish (Zv8) as the source genome and mouse (m37) as the outgroup [24,25]. The strongest synteny (greatest number of orthologous gene pairs) occurred between the segments of zebrafish chromosome 3 and mouse chromosome 7 that contain the *kcnc3a *and *Kcnc3 *genes, respectively. The approximate positions of *kcnc3a *and *Kcnc3 *are marked in red. Other orthologous pairs of genes are connected by lines. Subclusters of genes identified during the same pass of the sliding window are indicated using lines of the same color. Non-paired genes are not shown. The figure depicts the relative locations of genes but is not drawn to physical scale. Synteny was also detected between the regions of zebrafish chromosome 24 and mouse chromosome 7 that contain *kcnc3b *and *Kcnc3*, respectively (data not shown).

To verify the identity of mammalian *KCNC *orthologs in zebrafish, we analyzed synteny between the zebrafish and mouse genomes using the online program Synteny Database [24,25]. The strongest syntenic clusters, containing the greatest number of orthologous gene pairs, were found between chromosomal segments containing the following pairs of zebrafish and mouse genes: *kcnc1b *and *Kcnc1*; *kcnc2 *and *Kcnc2*; *kcnc3a *and *Kcnc3; *and *kcnc4 *and *Kcnc4 *(Fig. 2 [*kcnc3a*/*Kcnc3 *pair] or data not shown). These results confirm the evolutionary relationship between genes encoding Kv3 family members in zebrafish and mammals.

**Figure 3:**
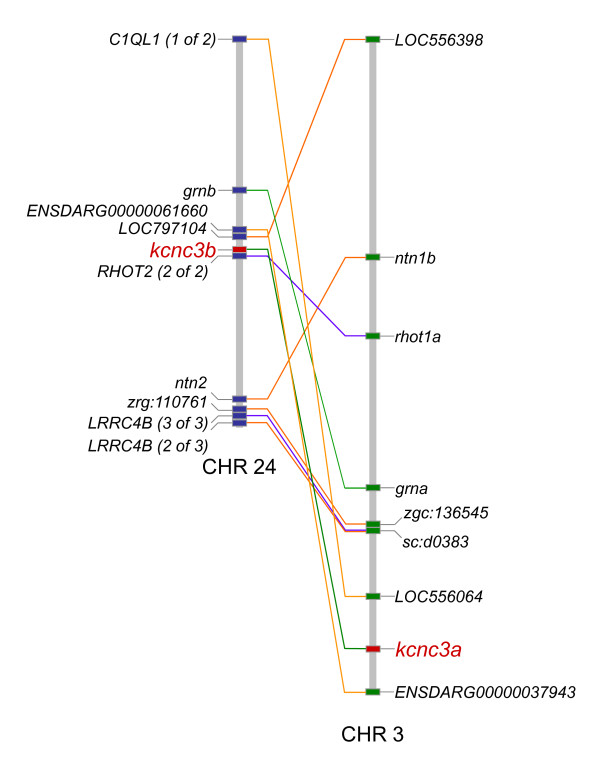
**Synteny between *kcnc3 *paralogs on zebrafish chromosomes 3 and 24**. Orthologous pairs of genes, including *kcnc3a *and *kcnc3b *on zebrafish chromosomes 3 and 24, respectively, are connected by lines. The approximate positions of *kcnc3a *and *kcnc3b *are marked in red. Synteny was detected and has been depicted as described in the legend to Fig. 2.

To verify the existence of paralogous *kcnc1 *and *kcnc3 *genes in zebrafish, we used Synteny Database to identify regions of conserved synteny on chromosomes 7 and 25 (*kcnc1a *and *kcnc1b*) and chromosomes 3 and 24 (*kcnc3a *and *kcnc3b*) (Fig. 3 [chromosome 3/24 pair] or data not shown) [24,25]. As expected, the chromosomal segments containing *kcnc1a*/*kcnc1b *and *kcnc3a*/*kcnc3b *pairs showed the strongest synteny in the zebrafish genome. The identification of two *kcnc1 *and two *kcnc3 *paralogs in zebrafish reflects genome duplication that occurred early during teleost evolution, after divergence of mammalian and teleost ancestors [26].

### The zebrafish genome contains two functional Kv3.3 paralogs

According to the Zv8 genome release, plausible Kv3 genes were located at five of the six identified genomic locations. Putative coding exons were encoded on a single strand and were located in the correct order along the chromosome (Table 1). In contrast, the Kv3.3 gene on chromosome 24, *kcnc3b*, was rearranged (Table 1). Because the genome assembly is preliminary, this does not preclude the presence of an intact Kv3.3 gene on chromosome 24.

**Figure 4:**
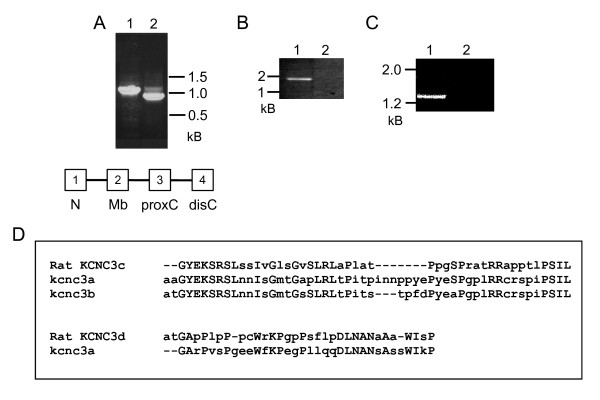
**Zebrafish contain two Kv3.3 genes**. A) Properly-spliced products of the expected sizes are amplified by RT-PCR using *kcnc3a*- and *kcnc3b*-specific primers. Lane 1: Product of *kcnc3a*-specific primers in exons 1 and 2 as indicated in cartoon below gel, which shows the conserved genomic arrangement of Kv3 protein coding exons 1 (N terminus), 2 (S1-S6 membrane domain), 3 (proximal C terminus), and 4 (distal C terminus) (Table 1). Cartoon is not drawn to scale. Lane 2: Product of *kcnc3b*-specific primers in exons 2 and 3. B), C) Mixed primers from *kcnc3a *and *kcnc3b *fail to amplify products. Lanes 1: In B, product of *kcnc3a*-specific primers in exons 1 and 4 (specific for 'PSIL' carboxyl terminus [see part D]); In C, product of *kcnc3b*-specific primers in exons 2 and 4. Lanes 2: In B, mixture of *kcnc3a*-specific primer in exon 1 and *kcnc3b-*specific primer in exon 4; In C, mixture of *kcnc3b*-specific primer in exon 2 and *kcnc3a*-specific primer in exon 4. Primer sequences are provided in additional file 2, Fig. S2. D) Distal carboxyl termini generated by alternative splicing of *kcnc3a *have been aligned with orthologous sequences from rat *Kcnc3 *gene. Identical residues are shown in caps. RT-PCR analysis indicates that both *kcnc3a *variants are expressed in embryonic zebrafish (data not shown). Both *kcnc3a *variants, ending in 'WIKP' or 'PSIL', were cloned for functional analysis. Only one exon encoding the distal carboxyl terminus of *kcnc3b *has been identified in Zv8. Accession numbers for rat splice variants: [GenBank:AY179603.1, GenBank:AY179604.1].

**Figure 5:**
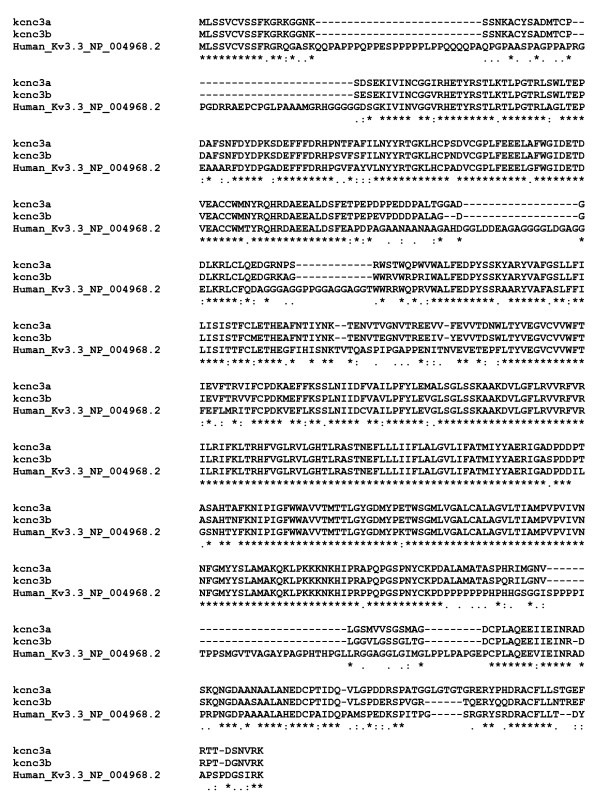
**Alignment of the predicted protein sequences of zebrafish and human Kv3.3**. Protein sequences of *kcnc3a*, *kcnc3b*, and human Kv3.3 encompassing the amino terminus, S1-S6 membrane domain, and proximal carboxyl terminus were aligned using Clustal W (1.81) [34]. Sequences of the distal carboxyl termini in *kcnc3a *and *kcnc3b *are provided in Fig. 4D. Accession numbers: *kcnc3a *sequence with 'WIKP' ending, [GenBank: HQ118212]; *kcnc3a *sequence with 'PSIL' ending, [GenBank: HQ118213]; *kcnc3b *sequence, [GenBank:HQ118214].

We used reverse transcriptase PCR (RT-PCR) to determine whether *kcnc3a *and *kcnc3b *are functional genes that are transcribed and appropriately spliced in zebrafish. RNA was extracted from embryos at 2 to 3 days post fertilization and amplified using gene-specific primers located in different exons. Each primer set yielded products of the expected size for a properly transcribed and spliced mRNA (Fig. 4A-C; additional file 2, Fig. S2). Using RT-PCR, we cloned and sequenced cDNAs corresponding to *kcnc3a *and *kcnc3b *amplified from embryonic cDNA or an adult retinal cDNA library. Their nucleic acid and predicted protein sequences matched the genomic sequences found on chromosomes 3 and 24, respectively (Additional file 1, Fig. S1; Fig. 5). The Kv3.3 paralogs are closely related. From the amino terminus through the proximal carboxyl terminus, the predicted amino acid sequences of *kcnc3a *and *kcnc3b *are 89% identical (Fig. 5). RT-PCR analysis indicated that *kcnc3a *mRNA is alternatively spliced, leading to protein variants with different carboxyl-terminal sequences (Fig. 4D). We conclude that there are two functional Kv3.3 genes in the zebrafish genome, despite the fact that one of them is not co-linear in the Zv8 genome release.

### Kv3 subfamily-specific gating properties are conserved in zebrafish Kv3.3

**Figure 6:**
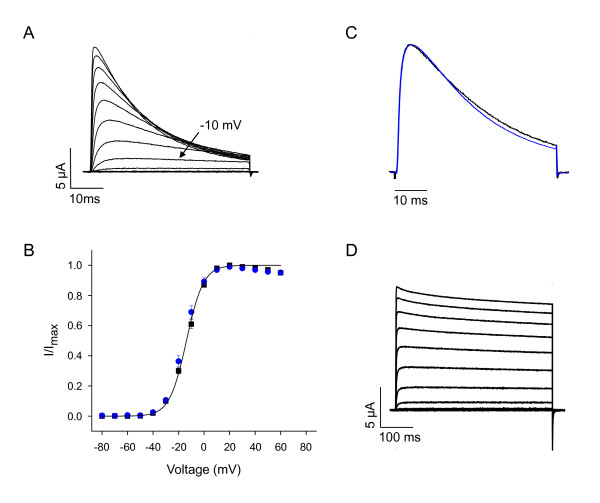
**Conserved gating properties in zebrafish Kv3.3 channels**. A) Wild type zebrafish Kv3.3 currents were evoked by pulsing from -90 mV to voltages ranging from -80 to +60 mV in 10 mV increments. The bath contained 4 mM K^+^. The -10 mV trace is labeled for comparison to Fig. 10A. The traces were obtained using the *kcnc3a *WIKP splice variant (see Fig. 4D). B) Normalized amplitudes of isochronal tail currents have been plotted versus voltage. Tail currents were recorded in an 89 mM Rb^+ ^bath solution. The membrane was pulsed from -90 to +60 mV for 15 ms prior to repolarization to -90 mV. Data were fitted with a single Boltzmann function to obtain values for the midpoint voltage (V_1/2_) and slope factor, which were -13.8 ± 0.3 mV and 6.6 ± 0.1 mV, respectively (*n *= 5). Black squares, *kcnc3a *WIKP splice variant; blue circles: *kcnc3a *PSIL splice variant (see Fig. 4D). C) Currents were evoked by pulsing from -90 mV to +20 mV. Black trace: *kcnc3a *WIKP variant; blue trace: *kcnc3a *PSIL variant (see Fig. 4D). Traces were scaled to the same amplitude and overlaid. D) The N-terminal extension of Kv3.3 between the first and second methionine residues was removed by mutating the initial ATG. Current traces were evoked by pulsing from -90 mV to voltages ranging from -80 to +60 mV in 10 mV increments. Currents are shown on a compressed time scale. The traces were obtained using the *kcnc3a *WIKP splice variant (see Fig. 4D).

To characterize the functional properties of zebrafish Kv3.3 channels, cDNA sequences encoding the two *kcnc3a *carboxyl-terminal splice variants were cloned into the Bluescript vector for in vitro transcription of RNA. RNA encoding each splice variant was separately injected into *Xenopus *oocytes for voltage clamp analysis (Fig. 6). Zebrafish Kv3.3 channels were robustly active with properties expected for a member of the Kv3 family [1,4]. Significant activation was detected starting at -20 mV (Fig. 6A). In contrast, in Shaker, a member of the Kv1 family, currents are typically detected between -50 and -40 mV. The probability of Kv3.3 channel opening, determined from normalized isochronal tail current amplitudes, increased steeply between -20 and 0 mV, with a midpoint voltage (V_1/2_) of -13.8 mV (Fig. 6B). Upon repolarization to the holding voltage, channels closed rapidly (see below). We conclude that the novel gating properties of Kv3 channels from higher vertebrates are conserved in zebrafish Kv3.3, consistent with a role in facilitating fast, repetitive firing in neurons.

Alternative splicing of the carboxyl terminus had no significant effect on channel function (Fig. 6B, C). Similarly, alternative splicing of mammalian Kv3 genes does not alter the functional properties of the channel [1,27]. Instead, different Kv3 carboxyl termini may be involved in targeting channel proteins to different subcellular compartments [27].

Zebrafish Kv3.3 currents showed prominent inactivation (Fig. 6A, C). Zebrafish channels inactivated more quickly than human Kv3.3 (data not shown), presumably because the amino terminal extension is shorter in the fish protein (Additional file 1, Fig. S1; Fig. 5) [28]. Deleting the amino terminal extension removed fast inactivation, indicating that zebrafish Kv3.3, like mammalian Kv3.3 channels, is subject to N-type ball and chain inactivation (Fig. 6D) [1,28]. Co-expression of the non-inactivating zebrafish Kv3.3 with wild type human Kv3.3 produced currents with intermediate inactivation kinetics, consistent with co-assembly of the human and zebrafish subunits into functional tetrameric channels, as expected (data not shown) [29].

**Figure 7:**
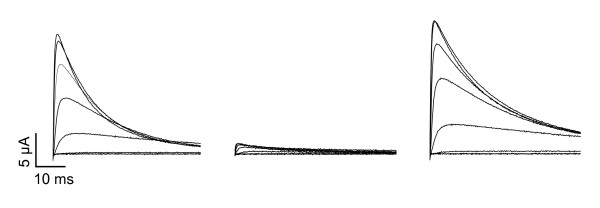
**TEA sensitivity of zebrafish Kv3.3 channels**. Zebrafish Kv3.3 currents were recorded in the absence (left) or presence (middle) of 1 mM TEA-Cl. TEA block is fully reversible (right, recorded after TEA washout). Currents were evoked by stepping from -90 mV to voltages ranging from -60 to +80 mV in 20 mV increments. The traces were obtained using the *kcnc3a *WIKP splice variant (see Fig. 4D).

Mammalian Kv3 channels are highly sensitive to the pore blocker tetraethylammonium (TEA) [1]. Unlike most K^+ ^channels, which have a lower affinity for TEA, mammalian Kv3 channels are nearly completely blocked by 1 mM TEA [1]. To investigate the TEA sensitivity of zebrafish Kv3.3 channels, currents were recorded in the presence and absence of 1 mM TEA (Fig. 7). At +80 mV, 1 mM TEA blocked 91% ± 1% of the Kv3.3 current amplitude (mean ± SEM, *n *= 4). We conclude that high sensitivity to TEA is conserved in zebrafish Kv3.3 channels.

### SCA13 mutations have conserved functional effects in human and zebrafish Kv3.3

**Figure 8:**
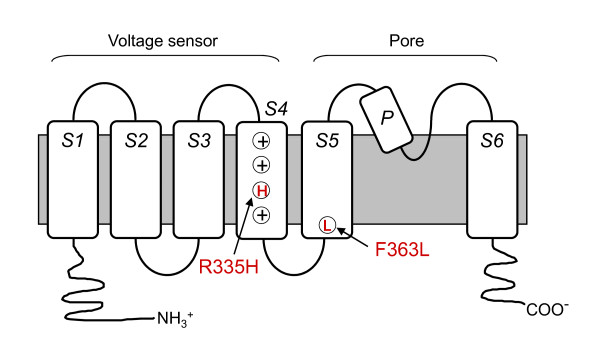
**Membrane topology of a Kv subunit showing SCA13 mutations**. The cartoon has been marked to indicate the approximate locations of the R335H and F363L mutations. The S1-S6 transmembrane segments, the re-entrant pore (P) loop, and the amino and carboxyl termini are labeled. Brackets denote the voltage sensor domain (S1-S4) and pore region (S5-P-S6). Circled plus signs in S4 indicate positively-charged arginine residues that sense voltage [35,36]. The third circle contains an H denoting the R335H mutation. Circled L in S5 indicates the location of the F363L mutation.

**Figure 9:**
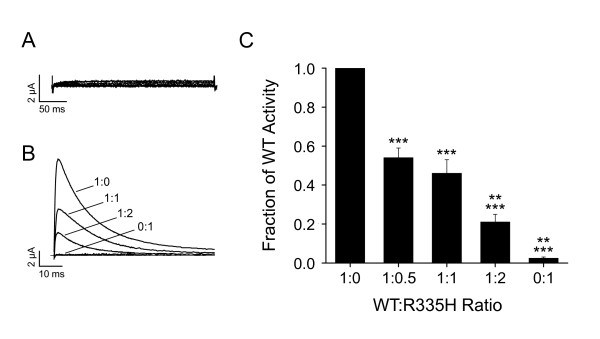
**Conserved effect of dominant negative SCA13 mutation inserted into zebrafish Kv3.3**. A) R335H RNA did not generate functional channels when injected alone into oocytes. The membrane was pulsed from a holding potential of -90 mV to voltages ranging from -80 to +60 mV in 10 mV increments. B) RNA for wild type zebrafish Kv3.3 (0.1 ng) and the R335H mutant were mixed in the indicated ratios and injected into oocytes. Representative current traces obtained from each mixture at +40 mV have been superimposed. C) The fraction of wild type current amplitude remaining has been plotted versus the injected ratio of wild type:R335H RNA. Amplitudes were measured at +40 mV. Means differed significantly by one way ANOVA (p < 0.0001) followed by Student's t-test, (1:0, *n *= 13; 1:0.5, *n *= 18; 1:1, *n *= 13; 1:2, n = 15; 0:1, *n *= 7). Pairwise comparisons: ***, significantly different from wild type alone (1:0), p < 0.001; **, significantly different from 1:1 ratio, p < 0.005.

To explore further the functional relationship between zebrafish and mammalian Kv3.3 channels, we introduced into *kcnc3a *two SCA13 mutations corresponding to R420H and F448L in human Kv3.3 (Fig. 8). Similarly to R420H, the corresponding mutation R335H was not functional when expressed alone but exerted a dominant negative effect on the activity of the zebrafish wild type subunit (Fig. 9). Because SCA13 is an autosomal dominant disease, the dominant negative effect of R420H, recapitulated in R335H, is likely essential to the etiology of adult onset ataxia and neurodegeneration in affected individuals [4].

**Figure 10:**
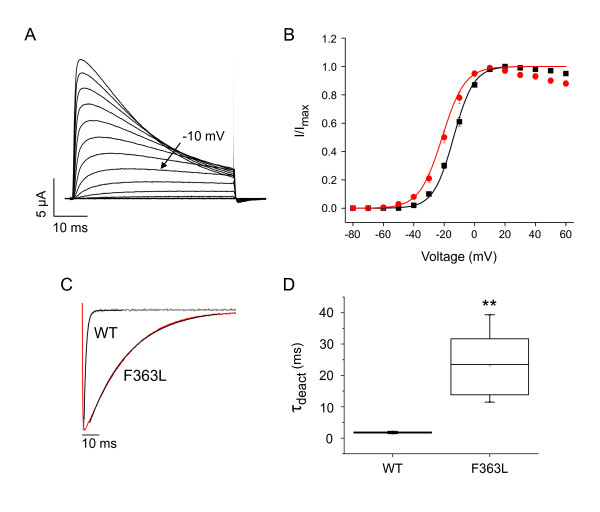
**Conserved effect of dominant gain of function gating mutation inserted into zebrafish Kv3.3**. A) F363L currents were evoked in oocytes by pulsing from a holding potential of -90 mV to voltages ranging from -80 to +60 mV in 10 mV increments. The bath contained 4 mM K^+^. The -10 mV trace is labeled for comparison to Fig. 6A. B) Normalized amplitudes of isochronal tail currents from F363L channels (red circles) have been plotted versus voltage. Data from wild type channels (black squares) are shown for comparison. Tail currents were recorded in an 89 mM Rb^+ ^bath solution. The membrane was pulsed from -90 to +60 mV for 15 ms prior to repolarization to -90 mV. Data were fitted with a single Boltzmann function to obtain values for the midpoint voltage (V_1/2_) and slope factor, which were -21.4 ± 0.6 mV and 7.4 ± 0.2 mV, respectively (*n *= 8). C) Tail currents from wild type (gray trace) and F363L (red trace) channels were evoked in an 89 mM Rb^+ ^bath solution by repolarizing from +40 to -90 mV. Tail current traces were fitted with a single exponential function (black curves). D) Box plot shows fitted values of τ_deact _at -90 mV. Mean τ_deact _values for wild type (*n *= 5) and F363L (*n *= 7) were 1.8 ± 0.1 ms (*n *= 5) and 23.3 ± 3.9 ms (*n *= 7), respectively. **, significantly different from wild type, p < 0.005 by Student's t-test.

F363L in *kcnc3a *corresponds to the human mutation F448L, which alters channel gating and causes infant onset motor problems and maldevelopment of the cerebellum [4]. F363L generated functional channels in oocytes. Significant activation was detected at -40 to -30 mV, indicating that the voltage dependence of pore opening was shifted in the hyperpolarized direction (Fig. 10A). The midpoint voltage for activation, determined from the amplitudes of normalized isochronal tail currents, was -21.4 mV, corresponding to a shift of -7.6 mV compared to wild type Kv3.3 (Fig. 10B). For comparison, the analogous mutation in human Kv3.3 (F448L) shifts activation ~12 mV in the hyperpolarized direction [4].

Similarly to the human mutation, F363L dramatically slowed channel deactivation (Fig. 10C). Tail currents, evoked in an 89 mM Rb^+ ^bath solution by repolarizing the membrane from +40 to -90 mV, were fitted with a single exponential function to characterize deactivation kinetics (Fig. 10D). Values of τ_deact _in wild type Kv3.3 and the F363L mutant were 1.8 ms and 23.3 ms, respectively. Thus, F363L slowed channel closing by ~13-fold at -90 mV. For comparision, the analogous mutation in human Kv3.3 (F448L) slowed channel closing by ~7-fold at -90 mV [4]. Therefore, like its human counterpart, the F363L mutation specifically affects the voltage dependence of activation and the kinetics of channel closing, converting the unique Kv3 gating properties to more closely resemble those of a Shaker-type channel [4]. Our results demonstrate that SCA13 mutations have similar effects on human and zebrafish Kv3.3 channels, confirming that there is strong functional homology between Kv3.3 in teleost and mammalian species [30].

## Conclusions

Genes encoding the voltage-dependent Kv3.1, Kv3.2, Kv3.3, and Kv3.4 K^+ ^channels have been identified in the zebrafish, *Danio rerio*. Two paralogous genes exist for both Kv3.1 and Kv3.3, reflecting an ancient genome duplication in the teleost line during evolution [26]. The unique gating properties of Kv3 channels are conserved in zebrafish Kv3.3, suggesting that Kv3 channels play an essential role in facilitating fast spiking in zebrafish neurons. SCA13 mutations have very similar effects on the activity of human and zebrafish Kv3.3 channels, indicating that the mutations affect fundamental channel properties that have been conserved during evolution. Our results suggest that expressing SCA13 mutant subunits in zebrafish will provide an animal model useful for investigating the cellular basis of SCA13.

## Methods

### Bioinformatic, phylogenetic, and syntenic analysis

The current Ensembl genome release (Zv8) was probed with mammalian Kv3 sequences using Tblastn to identify coding exons, which were assembled and translated. Sequences were aligned with the sequences of Kv3 family members from mammalian, amphibian, and teleost species using the program Muscle v3.7 and the alignment was manually adjusted (Additional file 1, Fig. S1) [20,21]. A phlyogenetic tree was constructed with the program PhyML using 100 bootstrapping replicates and the Jones, Taylor, Thornton amino acid substitution model [22,23]. Synteny was evaluated for each Kv3 family member using the program Synteny Database with zebrafish (release Zv8) as the source genome, mouse (release m37) as the outgroup, and a sliding window size of 100 genes [24,25]. This program is designed for analyzing genomes that have undergone complete duplication during evolution. It identifies pairwise clusters of orthologs and paralogs simultaneously.

### Cloning and mutagenesis

Full length *kcnc3a *cDNA clones with the WIKP or PSIL alternatively spliced carboxyl termini were cloned by RT-PCR using gene specific primers designed from Zv8 genomic sequences (Fig. 4D). Amplified sequences were inserted into Bluescript. Mutations were generated in the WIKP splice variant using the QuikChange method (Stratagene, La Jolla, CA). Mutations were verified by sequencing. Linearized plasmid DNA was transcribed using the mMessage mMachine T7 or T7 Ultra kit (Ambion, Austin, TX). RNA for each splice variant was separately injected into *Xenopus *oocytes using standard methods [31,32].

### Electrophysiology

One to two days after RNA injection, ionic currents were recorded at room temperature (20-22°C) using a Warner OC-725C two electrode voltage clamp [31,32]. Electrodes were filled with 3 M KCl and had resistances of 0.3-1.0 MΩ. Oocytes were bathed in 85 mM NaCl, 4 mM KCl, 1.8 mM CaCl_2_, 10 mM HEPES, pH 7.2. To record tail currents, 85 mM NaCl +4 mM KCl was replaced by 89 mM RbCl. Pulse protocols were generated and data were acquired using pClamp software (Axon Instruments, Foster City, CA). Data were sampled at 10 kHz and filtered at 2 kHz using an 8-pole Bessel filter (Frequency Devices, Haverhill, MA). Linear capacitive and leak currents were subtracted using a P/-4 protocol [33].

Currents were evoked by pulsing from a holding potential of -90 mV to voltages ranging from -80 mV to +60 mV in 10 mV increments. The probability of channel opening as a function of voltage was determined from isochronal tail current amplitudes. Tail current amplitudes were normalized by the maximum value obtained in the experiment and plotted versus voltage. Data were fitted with a single Boltzmann function to determine values for the midpoint potential (V_1/2_) and slope factor. Deactivation kinetics were measured from tail currents evoked by pulsing to +40 mV before repolarizing to -90 mV. Tail currents were fitted with a single exponential function to obtain values for the deactivation time constant (τ_deact_).

## Abbreviations

SCA13: spinocerebellar ataxia type 13; RT-PCR: reverse transcriptase-polymerase chain reaction; TEA: tetraethylammonium ion

## Competing interests

The authors declare that they have no competing interests.

## Authors' contributions

AFM and GR cloned and sequenced cDNAs for *kcnc3a *and *kcnc3b*. JLR and JYH carried out the voltage clamp experiments. DMP conceived of, designed and directed the study; analyzed data; carried out bioinformatic, phylogenetic, and syntenic analyses; and wrote the manuscript. All authors read and approved the final manuscript.

## Supplementary Material

Additional file 1 Fig. S1: Alignment of zebrafish Kv3 sequences with 10 Kv3 sequences from other species. Click here for file

Additional file 2 Fig. S2: Primer sequences used in PCR experiments shown in Fig. 4 Click here for file
