# Supplementary material for: Functional effects of spinocerebellar ataxia type 13 mutations are conserved in zebrafish Kv3.3 channels
Source: BMC Neurosci. 2010 Aug 16;11:99. doi: 10.1186/1471-2202-11-99 (PMC2933717; doi:10.1186/1471-2202-11-99)
Supplement: Additional file 1 — Fig. S1: Alignment of zebrafish Kv3 sequences with 10 Kv3 sequences from other species. [file 1471-2202-11-99-S1.PDF]

SUPPLEMENTAL FIGURE S1

Figure S1. Alignment of zebrafish Kv3 sequences with 10 Kv3 sequences from other species (h, human; m, mouse; Xen, *Xenopus*; Apt, *Apteronotus*). Protein sequences encompassing the amino terminus, S1-S6 membrane domain, and proximal carboxyl terminus were aligned using Muscle 3.7 and the alignment was manually adjusted. Accession numbers for non-zebrafish Kv3 sequences are given in the figure.

```
>kcnc2
----------------------------------------------------------------------------------
-MGK----FDERERVILNVGGTRHETYRSTLKTLPGTRLALLAS-ESDLELVLDQLQQV--------------------PGF
IEYSARSN---------------EYFFDRHPGVFAYVLNYYRTGKLHCPADVCGPLFEEELSFWGIDETDVEPCCWMTYRQH
RDAEEALDVFEMNV--------------DTEEDDEYG----------------KRLGIED---VVSADGTVS--------RW
RRWQPVIWNLFDDPYSSRAARFIAFASLFFIMVSITTFCLETHEAFNTIINKTDPLRN---ETLTDISQQYEIETDPALTYV
EGVCVLWFTFEFLVRITFSPDKLEFIKSVLNIIDFVAILPFYLEVGLSGLSSKAAKDVLGFLRVVRFVRILRIFKLTRHFVG
LRVLGHTLRASTNEFLLLIIFLALGVLIFATMIYYAERIGASPNDP-TASHHTMFKNIPIGFWWAVVTMTTLGYGDMYPQTW
SGMVVGALCALAGVLTIAMPVPVIVNNFGMYYSLAMAKQKLPKKRKKHIPQAVQ-TGSPLYCRTD-----LSTAC-------
--------------------------NSTQ----------------------GELCIG--QSRGL-ERHRSGETLVH--LHA
VLSADCSAGSDISM----SPEERMP----MRHSSVREQDHHTEGTCFLLAPSDYTCTADAAIRK
>Human_Kv3.2a_NP_631874.1
----------------------------------------------------------------------------------
-MGK----IENNERVILNVGGTRHETYRSTLKTLPGTRLALLASSEPPGDCLTTAGDKLQPSPPPLSPPPRAPPLSPGPGGC
FEGGAGNCSSRGGRASDHPGGGREFFFDRHPGVFAYVLNYYRTGKLHCPADVCGPLFEEELAFWGIDETDVEPCCWMTYRQH
RDAEEALDIFETPD-----------LIGGDPGDDEDLAA--------------KRLGIEDAAGLGGPDGK-SG-------RW
RRLQPRMWALFEDPYSSRAARFIAFASLFFILVSITTFCLETHEAFNIVKNKTEPVIN-----GTSVVLQYEIETDPALTYV
EGVCVVWFTFEFLVRIVFSPNKLEFIKNLLNIIDFVAILPFYLEVGLSGLSSKAAKDVLGFLRVVRFVRILRIFKLTRHFVG
LRVLGHTLRASTNEFLLLIIFLALGVLIFATMIYYAERVGAQPNDP-SASEHTQFKNIPIGFWWAVVTMTTLGYGDMYPQTW
SGMLVGALCALAGVLTIAMPVPVIVNNFGMYYSLAMAKQKLPRKRKKHIPPAPQ-ASSPTFCKTE-----LNMAC-------
--------------------------NSTQ----------------------SDTCLG-KDNRLL-EHNR-----------S
VLSGDDSTGSEPPL----SPPERLP----IRRSSTRDKNRRGE-TCFLLTTGDYTCASDGGIRK
>Mouse_Kv3.2_NP_001020752.1
----------------------------------------------------------------------------------
-MGK----IESNERVILNVGGTRHETYRSTLKTLPGTRLALLASSEPQGDCLTAAGDKLQPLPPPLSPPPRPPPLSPVPSGC
FEGGAGNCSSHGGNGGDHPGGGREFFFDRHPGVFAYVLNYYRTGKLHCPADVCGPLFEEELAFWGIDETDVEPCCWMTYRQH
RDAEEALDIFETPD-----------LIGGDPGDDEDLAA--------------KRLGIEDAAGLGGPDGK-SG-------RW
RKLQPRMWALFEDPYSSRAARFIAFASLFFILVSITTFCLETHEAFNIVKNKTEPVIN-----GTSPVLQYEIETDPALTYV
EGVCVVWFTFEFLVRIVFSPNKLEFIKNLLNIIDFVAILPFYLEVGLSGLSSKAAKDVLGFLRVVRFVRILRIFKLTRHFVG
LRVLGHTLRASTNEFLLLIIFLALGVLIFATMIYYAERVGAQPNDP-SASEHTQFKNIPIGFWWAVVTMTTLGYGDMYPQTW
SGMLVGALCALAGVLTIAMPVPVIVNNFGMYYSLAMAKQKLPRKRKKHIPPAPL-ASSPTFCKTE-----LNMAC-------
--------------------------NSTQ----------------------SDTCLG-KENRLL-EHNR-----------S
VLSGDDSTGSEPPL----SPPERLP----IRRSSTRDKNRRGE-TCFLLTTGDYTCASDGGIRK
>kcnc4
MISSVCVSSY-RGRKSGNKP------------------------------------------------------PSKSCLKE
EMAR----CEDSDKIIINVGGTRHETYRSTLRTIPGSRLAWLA--DTDSQVNPDADTGQTHPNS------------------
-------------------NGGSEFFFDRHPGIFAYVLNYYRTGKLHCPADVCGPLFEEELAFWGIDETDVEPCCWMTYRQH
RDAEEALDIFEPPD-------------PEDAEDDQ---------------DMPRRFGIED-----SPD-RSRG-------CC
EVWQPKIWALFEDPYSSRAARVIAFVSLFFILVSITNFCLETHEAFFEFHNHTDTKSA---TNSTTIIQVMD--TKPVLTVV
EGICVVWFTFEFLVRIICCPNKLLFIKNQLNIIDFVAILPFYLERSLIGNTSKAAKDVLGFLRVVRFVRILRIFKLTRHFVG
LRVLGHTLRASVNEFCLLIIFLALGVLIFATMIYYAERISADPDDP-SGCNHTHFKNIPIGFWWAVVTMTTLGYGDMVPKTW
LGMIVGALCAIAGVLTIAMPVPVIVNNFGMYYSLAMAKQKLPKKKKKHNPNPGMVLDSGSFGKSE-----SNSPR-------
--------------------------NSTQ----------------------SDTCPL-AAEENIT-RNRSDSKQNGD-ATV
TLSEEEGCSLTQPL----SPGERWT----LRCSRNRDKTMKDA-TCFLLNSGDFSCGAEPTIHT
>Mouse_Kv3.4_NP_666034.1
MISSVCVSSY-RGRKSGNKP------------------------------------------------------PSKTCLKE
EMAK----GEASEKIIINVGGTRHETYRSTLRTLPGTRLAWLA--DPDGGGRPESDGGGAGSSGSSGGGGG-----------
-------------------GGGCEFFFDRHPGVFAYVLNYYRTGKLHCPADVCGPLFEEELTFWGIDETDVEPCCWMTYRQH
RDAEEALDIFESPD-----GGGGGAGPGDEAGDDERELAL-------------QRLGPHE---GGSGPGAGSG-------GC
RGWQPRMWALFEDPYSSRAARVVAFASLFFILVSITTFCLETHEAFNIDRNVTEIHRV---GNITSVRFRREVETEPILTYI
EGVCVMWFTLEFLVRIVCCPDTLDFVKNLLNIIDFVAILPFYLEVGLSGLSSKAARDVLGFLRVVRFVRILRIFKLTRHFVG
LRVLGHTLRASTNEFLLLIIFLALGVLIFATMIYYAERIGARPSDP-RGNDHTDFKNIPIGFWWAVVTMTTLGYGDMYPKTW
SGMLVGALCALAGVLTIAMPVPVIVNNFGMYYSLAMAKQKLPKKRKKHVPRPPQ-LESPIYCKSE-----ETSPR-------
--------------------------DSTY----------------------SDTSPP-AREEGVVERKRADSKQNGD-ANA
VLSDEEGAGLTQPLALAPTPEERRA----LRRSGTRDRNKKAA-ACFLLSAGDYAC-ADGSVRK
>Human_Kv3.4c_NP_001034663.1
MISSVCVSSY-RGRKSGNKP------------------------------------------------------PSKTCLKE
EMAK----GEASEKIIINVGGTRHETYRSTLRTLPGTRLAWLA--DPDGGGRPETDGGGVGSSGSSG---------------
-------------------GGGCEFFFDRHPGVFAYVLNYYRTGKLHCPADVCGPLFEEELTFWGIDETDVEPCCWMTYRQH
RDAEEALDIFESPD-----GGGSGAGPSDEAGDDERELAL-------------QRLGPHE---GGAGHGAGSG-------GC
RGWQPRMWALFEDPYSSRAARVVAFASLFFILVSITTFCLETHEAFNIDRNVTEILRV---GNITSVHFRREVETEPILTYI
```

```
EGVCVLWFTLEFLVRIVCCPDTLDFVKNLLNIIDFVAILPFYLEVGLSGLSSKAARDVLGFLRVVRFVRILRIFKLTRHFVG
LRVLGHTLRASTNEFLLLIIFLALGVLIFATMIYYAERIGARPSDP-RGNDHTDFKNIPIGFWWAVVTMTTLGYGDMYPKTW
SGMLVGALCALAGVLTIAMPVPVIVNNFGMYYSLAMAKQKLPKKRKKHVPRPAQ-LESPMYCKSE-----ETSPR-------
--------------------------DSTC----------------------SDTSPP-AREEGMIERKRADSKQNGD-ANA
VLSDEEGAGLTQPLASSPTPEERRA----LRRSTTRDRNKKAA-ACFLLSTGDYAC-ADGSVRK
>kcnc1b
----------------------------------------------------------------------------------
-MGQ----GDDKDRIVINVGGIKHQTYRSTLRTLPGTRLSWLA--EPDAPNNFDYDANIG----------------------
-----------------------EFFFDRHPSVFAHILNYYRTGKLHCPADVCGPLYEEELAFWGIDETDVEPCCWMTYRQH
REAEEALDSF-------------TGGALDLGHDDPEPEGVVEAAEGDEGVEMTRRLAQGD-----SPDNR-SG-------RW
SRWQKKAWALFEDPYSSKYARWVAFASLFFILVSITTFCLETHEAFNPIINRTETYME---GNETVERIFPETETMVQLTYI
EGVCVVWFTFEFIIRVTTCPDKLKFLRNTLNIIDFVAILPFYLEVGLSGLSSKAAKDVLGFLRVVRFVRILRIFKLTRHFVG
LRVLGHTLRASTNEFILLIIFLALGVLIFATMIYYAERIGRNPNDP-DASSDTHFKNIPIGFWWAVVTMTTLGYGDMYPQTT
SGMLVGALCALAGVLTIAMPVPVIVNNFGMYYSLAMAKQKLPKKKNKHIRRPPL-LGSPNYCRSA-----VNSPRPSTHSPK
KKNKHIRRPPLLGSPNYCRSAVNSPRPSTH----------------------SDTCPL-AQEEVS-EI-RYDFKVNGEPSKA
ALANEDCPHIDQAV----SPEE--V----FSPVD-RER------PCFLLTGGERANHTGGRVRK
>Human_Kv3.1a_NP_001106212.1
----------------------------------------------------------------------------------
-MGQ----GDESERIVINVGGTRHQTYRSTLRTLPGTRLAWLA--EPDAHSHFDYDPRAD----------------------
-----------------------EFFFDRHPGVFAHILNYYRTGKLHCPADVCGPLYEEELAFWGIDETDVEPCCWMTYRQH
RDAEEALDSF-------------GGAPLDNSADDADADGPGDSGDGEDELEMTKRLALSD-----SPDGRPGG-------FW
RRWQPRIWALFEDPYSSRYARYVAFASLFFILVSITTFCLETHERFNPIVNKTEIENV---RNGTQVRYYREAETEAFLTYI
EGVCVVWFTFEFLMRVIFCPNKVEFIKNSLNIIDFVAILPFYLEVGLSGLSSKAAKDVLGFLRVVRFVRILRIFKLTRHFVG
LRVLGHTLRASTNEFLLLIIFLALGVLIFATMIYYAERIGAQPNDP-SASEHTHFKNIPIGFWWAVVTMTTLGYGDMYPQTW
SGMLVGALCALAGVLTIAMPVPVIVNNFGMYYSLAMAKQKLPKKKKKHIPRPPQ-LGSPNYCKSV-----VNSPH-------
--------------------------HSTQ----------------------SDTCPL-AQEEIL-EINRADSKLNGEVAKA
ALANEDCPHIDQAL----TPDEGLP----FTRSGTRERY----GPCFLLSTGEYACPPGGGMRK
>Mouse_Kv3.1b_NP_032447.mod
----------------------------------------------------------------------------------
-MGQ----GDESERIVINVGGTRHQTYRSTLRTLPGTRLAWLA--EPDAHSHFDYDPRAD----------------------
-----------------------EFFFDRHPGVFAHILNYYRTGKLHCPADVCGPLYEEELAFWGIDETDVEPCCWMTYRQH
RDAEEALDSF-------------GGAPLDNSADDADADGPGDSGDGEDELEMTKRLALSD-----SPDGRPGG-------FW
RRWQPRIWALFEDPYSSRYARYVAFASLFFILVSITTFCLETHERFNPIVNKTEIENV---RNGTQVRYYREAETEAFLTYI
EGVCVVWFTFEFLMRVVFCPNKVEFIKNSLNIIDFVAILPFYLEVGLSGLSSKAAKDVLGFLRVVRFVRILRIFKLTRHFVG
LRVLGHTLRASTNEFLLLIIFLALGVLIFATMIYYAERIGAQPNDP-SASEHTHFKNIPIGFWWAVVTMTTLGYGDMYPQTW
SGMLVGALCALAGVLTIAMPVPVIVNNFGMYYSLAMAKQKLPKKKKKHIPRPPQ-LGSPNYCKSV-----VNSPH-------
--------------------------HSTQ----------------------SDTCPL-AQEEIL-EINRADSKLNGEVAKA
ALANEDCPHIDQAL----TPDEGLP----FTRSGTRERY----GPCFLLSTGEYACPPGGGMRK
>kcnc1a
----------------------------------------------------------------------------------
-MGQ----GDEKDRVVINVGGIRHQTYRSTLRTLPGTRLAWLA--EPDAHSHFDYDAQID----------------------
-----------------------EFFFDRHPGVFAHILNYYRTGKLHCPADVCGPLYEEELAFWGIDETDVEPCCWMTYRQH
REAEEALDSF-------------GGGPVEMGNDDMDTEALGDPGDGDEELEMTKRLAVGD-----SPDTKGAG-------FW
QRWQRRVWALFEDPYSSKYARWVAFASLFFILVSITTFCLETHEAFNPIINRTYVNTQ---DNTS--RFHLETETVVYLTYI
EGVCVVWFTFEFLMRVTFCPDKKKFIKNTLNIIDFVAILPFYLEVGLSGLSSSEAKDVLGFLRVVRFVRILRIFKLTRHFVG
LRVLGHTLRASTNEFLLLIIFLALGVLIFATMIYYAERIGANPNDP-RASEHTHFKNIPIGFWWAVVTMTTLGYGDMYPQTW
SGMLVGALCALAGVLTIAMPVPVIVNNFGMYYSLAMAKQKLPKKKNKHIPRAPQ-LGSANYCKSA-----MNSPH-------
--------------------------HSPQ---------------------ISEHCALAAQEEIL-EMNRADPKVNGEAAKA
ALANEDCPHIDQAI----SPEDGQI----FNPNEPRGDT-----PCFLLNVGRRSTNTGTRVRK
>Human_Kv3.3_NP_004968.2
MLSSVCVSSF-RGRQGASKQQP--APPPQPPESPPPPPLPPQQQQPAQPGPAASPAGPPAPRGPGDRRAEPCPGLPAAA---
-MGRHGGGGGDSGKIVINVGGVRHETYRSTLRTLPGTRLAGLT--EPEAAARFDYDPGAD----------------------
-----------------------EFFFDRHPGVFAYVLNYYRTGKLHCPADVCGPLFEEELGFWGIDETDVEACCWMTYRQH
RDAEEALDSFEAPDPAGAANAANAAGAHDGGLDDEAGAGGG-GLDGAG--GELKRLCFQDA--GGGAGGPPGGAGGAGGTWW
RRWQPRVWALFEDPYSSRAARYVAFASLFFILISITTFCLETHEGFIHISNKTVTQASPIPGAPPENITNVEVETEPFLTYV
EGVCVVWFTFEFLMRITFCPDKVEFLKSSLNIIDCVAILPFYLEVGLSGLSSKAAKDVLGFLRVVRFVRILRIFKLTRHFVG
LRVLGHTLRASTNEFLLLIIFLALGVLIFATMIYYAERIGADPDDI-LGSNHTYFKNIPIGFWWAVVTMTTLGYGDMYPKTW
SGMLVGALCALAGVLTIAMPVPVIVNNFGMYYSLAMAKQKLPKKKNKHIPRPPQ-PGSPNYCKPDPPPPPPPHPH-----HG
SGGISPPPPITPPSMGVTVAGAYPAGPHTHPGLLRGGAGGLGIMGLPPLPAPGEPCPL-AQEEVI-EINRADPRPNGDPAAA
ALAHEDCPAIDQPAM---SPEDKSP----ITPGS-RGRYSRDR-ACFLLT--DYAPSPDGSIRK
>Mouse_Kv3.3_NM_008422.2
```

```
MLSSVCVWSF-RGRQGTGKQQPQPVPTPQPPESSPPPLPPPQQQQCSQPGTAASPAGAPLSCGPGGRRAEPCPGLPAVA---
-MGRHGGGGGDSGKIVINVGGVRHETYRSTLRTLPGTRLAGLT--EPEAAARFDYDPGTD----------------------
-----------------------EFFFDRHPGVFAYVLNYYRTGKLHCPADVCGPLFEEELGFWGIDETDVEACCWMTYRQH
RDAEEALDSFEAPDSSANAN-ANAGGAHDAGLDDEAGAGGG-GLDGAG--GELKRLCFQDA--GGGAGGPAGGAGGAGGTWW
RRWQPRVWALFEDPYSSRAARYVAFASLFFILISITTFCLETHEGFIHISNKTVTQASPIPGAPPENITNVEVETEPFLTYV
EGVCVVWFTFEFLMRVTFCPDKVEFLKSSLNIIDCVAILPFYLEVGLSGLSSKAAKDVLGFLRVVRFVRILRIFKLTRHFVG
LRVLGHTLRASTNEFLLLIIFLALGVLIFATMIYYAERIGADPDDI-LGSNHTYFKNIPIGFWWAVVTMTTLGYGDMYPKTW
SGMLVGALCALAGVLTIAMPVPVIVNNFGMYYSLAMAKQKLPKKKNKHIPRPPQ-PGSPNYCKPDPPPPPPPHPH-----HG
SGGISPPPPITPPSMGVNVAGAYPPGPHTHPGLLRGGAGGLGIMGLPPLPAPGEPCPL-AQEEVI-ETNRADPRPNGDPAAA
ALAHEDCPAIDQPAM---SPEDKSP----ITPGS-RGRYSRDR-ACFLVT--DYAPSPDGSIRK
>Xenopus_Kv3.3_NP_001079081.1
----------------------------------------------------------------------------------
-MGK----NEDSDKIVINVGGIRHETYRSTPKTLPGTRLSWLT--EPDAFSNFDYDPKTD----------------------
-----------------------EFFFDRHPQVFACVLNYYRTGKLHCPSDVCGPLYEEELAFWGIDETDVEACCWMNYRQH
RDAEEALDSFETPEP----------------EEEED--------------GDLKRLCLQE-------DGRKLG-------WW
KRLRPKVWALFEDPYSSKYARYIALASLFFILISITTFCLETHEAFNDVNNKTEVFTQ---GNITKTETILEMETAPFLNYV
EGICVIWFTFEFLIRVIFCPDKMEFIKSSLNIIDFVAILPFYLEIGLSGLSSKAAKDVLGFLRVVRFVRILRIFKLTRHFVG
LRVLGHTLRASTNEFLLLIIFLALGVLIFATMIYYAERIGADPDDI-TGSKHTYFKNIPIGFWWAVVTMTTLGYGDMYPMTW
SGMLVGALCALAGVLTIAMPVPVIVNNFGMYYSLAMAKQKLPKKKNKHIPRPPL-PGSPNYCKPD-----LQSPH-------
--------------------------RSAQ----------------------GDACPL-AQEEII-EINRADSKQNGDAANA
ALANEDCPTIDQAL----SPEEKSP----ITPGG-RERYNRDR-ACFLLTTGDFAHSPDGNIRK
>kcnc3a
MLSSVCVSSF-KGRKGGNKS------------------------------------------------------SNKACYSA
DMTC----PSDSEKIVINCGGIRHETYRSTLKTLPGTRLSWLT--EPDAFSNFDYDPKSD----------------------
-----------------------EFFFDRHPNTFAFILNYYRTGKLHCPSDVCGPLFEEELAFWGIDETDVEACCWMNYRQH
RDAEEALDSFETPEP-------------DPPEDDPALTGGAD--------GDLKRLCLQE-------DGRNPS-------RW
STWQPWVWALFEDPYSSKYARYVAFGSLLFILISISTFCLETHEAFNTIYNKTENVTV---GNVTREEVVFEVVTDNWLTYV
EGVCVVWFTIEVFTRVIFCPDKAEFFKSSLNIIDFVAILPFYLEMALSGLSSKAAKDVLGFLRVVRFVRILRIFKLTRHFVG
LRVLGHTLRASTNEFLLLIIFLALGVLIFATMIYYAERIGADPDDP-TASAHTAFKNIPIGFWWAVVTMTTLGYGDMYPETW
SGMLVGALCALAGVLTIAMPVPVIVNNFGMYYSLAMAKQKLPKKKNKHIPRAPQ-PGSPNYCKPDALAMATASPH-------
------------RIMG-NVLGSMVVSGSMA----------------------GD-CPL-AQEEII-EINRADSKQNGDAANA
ALANEDCPTIDQVL----GPDDRSPATGGLGTGTGRERYPHDR-ACFLLSTGEFRT-TDSNVRK
>Apteronotus_Kv3.3_AF308934.1
MLSSVCVSSISKGRKGGNKS------------------------------------------------------SNKACYSA
DMPC----PSESEKIVINCGGVRHETYRSTLKTLPGTRLSWLT--EPDAFSNFDYDPKSD----------------------
-----------------------EFFFDRHPAVFSFILNYYRTGKLHCPNDVCGPLFEEELPFWGIDETDVEACCWMNYRQH
RDAEEALDSFETPEP-------------DAPEDDQALTGGAD--------GDLKRLCLQE-------DGRKVG-------WW
RVWQPRIWALFEDPYSSKYARYVAFGSLLFILISISTFCMETHEAFNTIYNKTENITV---GNVTREEIVYEVVTDSWLTYV
EGVCVIWFTIEVFMRVTFCPDKAEFFKSLLNIIDFVAILPFYLEVALSGLSSKAAKDVLGFLRVVRFVRILRIFKLTRHFVG
LRVLGHTLRASTNEFLLLIIFLALGVLIFATMIYYAERIAGNPDDPHRPARHTNFKNIPIGFWWAVVTMTTLGYGDMYPETW
SGMLVGALCALAGVLTIAMPVPVIVNNFGMYYSLAMAKQKLPKKKNKHIPRAPQ-PGSPNYCKPDALAMATASPQ-------
------------GILG-NVLGGVIGSAGLT----------------------GD-CPL-AQEEII-EINR-DSKQNGDAASA
ALADEDCPTIDQVL----SPDERSP----V--GRTRERYQQDR-ACFLLNTREFRP-ADGNVRK
>kcnc3b
MLSSVCVSSF-KGRKGGNKS------------------------------------------------------SNKACYSA
DMTC----PSESEKIVINCGGVRHETYRSTLKTLPGTRLSWLT--EPDAFSNFDYDPKSD----------------------
-----------------------EFFFDRHPSVFSFILNYYRTGKLHCPNDVCGPLFEEELAFWGIDETDVEACCWMNYRQH
RDAEEALDSFETPEP-------------EVPDDDPALAG--D--------GDLKRLCLQE-------DGRKAG-------WW
RVWRPRIWALFEDPYSSKYARYVAFGSLLFILISISTFCMETHEAFNTIYNKTENVTE---GNVTREEIVYEVVTDSWLTYV
EGVCVVWFTIEVFTRVVFCPDKMEFFKSPLNIIDFVAVLPFYLEVGLSGLSSKAAKDVLGFLRVVRFVRILRIFKLTRHFVG
LRVLGHTLRASTNEFLLLIIFLALGVLIFATMIYYAERIGASPDDP-TASAHTNFKNIPIGFWWAVVTMTTLGYGDMYPETW
SGMLVGALCALAGVLTIAMPVPVIVNNFGMYYSLAMAKQKLPKKKNKHIPRAPQ-PGSPNYCKPDALAMATASPQ-------
------------RILG-NVLGGVLGSSGLT----------------------GD-CPL-AQEEII-EINR-DSKQNGDAASA
ALANEDCPTIDQVL----SPDERSP----V--GRTQERYQQDR-ACFLLNTREFRP-TDGNVRK
```
