# Supplementary material for: Functional effects of spinocerebellar ataxia type 13 mutations are conserved in zebrafish Kv3.3 channels
Source: BMC Neurosci. 2010 Aug 16;11:99. doi: 10.1186/1471-2202-11-99 (PMC2933717; doi:10.1186/1471-2202-11-99)
Supplement: Additional file 2 — Fig. S2: Primer sequences used in PCR experiments shown in Fig. 4 [file 1471-2202-11-99-S2.PDF]

Figure 4	*Gene(s)*	*Upstream Primer*	*Downstream Primer*	*Locations*
Part A, lane 1	*kcnc3a*	5’- ACGTACCGCAGCACACTGAAGAC	5’-TTACAATGACGGGCACAGGCATGG	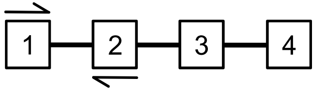
Part A, lane 2	*kcnc3b*	5’-CCATCTACAACAAGACGGAGAACG	5’-GTTGGACAGTCCTCGTTTGCTAGA	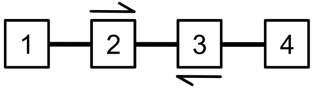
Part B, lane 1	*kcnc3a*	5’- ACGTACCGCAGCACACTGAAGAC	5’-CTACAGAATGGAGGGTATAGGGGAT	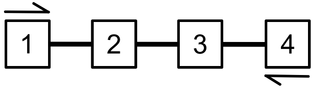
Part B, lane 2	*kcnc3a/3b*	5’- ACGTACCGCAGCACACTGAAGAC	5’-CTACAAAATGGAGGGAATGGGGGAG	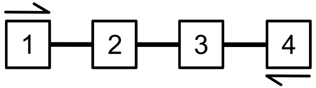
Part C, lane 1	*kcnc3b*	5’-CCATCTACAACAAGACGGAGAACG	5’-CTACAAAATGGAGGGAATGGGGGAG	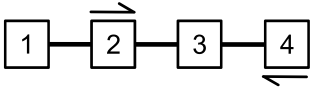
Part C, lane 2	*kcnc3b/3a*	5’-CCATCTACAACAAGACGGAGAACG	5’-CTACAGAATGGAGGGTATAGGGGAT	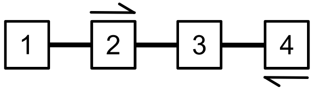
